# Transcriptome profile analysis of flowering molecular processes of early flowering trifoliate orange mutant and the wild-type [*Poncirus trifoliata *(L.) Raf.] by massively parallel signature sequencing

**DOI:** 10.1186/1471-2164-12-63

**Published:** 2011-01-26

**Authors:** Jin-Zhi Zhang, Xiao-Yan Ai, Lei-Ming Sun, Dong-Liang Zhang, Wen-Wu Guo, Xiu-Xin Deng, Chun-Gen Hu

**Affiliations:** 1 Key Laboratory of Horticultural Plant Biology (Ministry of Education), College of Horticulture and Forestry Science, Huazhong Agricultural University, Wuhan 430070, PR China; 2 National Key Laboratory of Crop Genetic Improvement, Huazhong Agricultural University, Wuhan, 430070, PR China

## Abstract

**Background:**

After several years in the juvenile phase, trees undergo flowering transition to become mature (florally competent) trees. This transition depends on the balanced expression of a complex network of genes that is regulated by both endogenous and environmental factors. However, relatively little is known about the molecular processes regulating flowering transition in woody plants compared with herbaceous plants.

**Results:**

Comparative transcript profiling of spring shoots after self-pruning was performed on a spontaneously early flowering trifoliate orange mutant (precocious trifoliate orange, *Poncirus trifoliata*) with a short juvenile phase and the wild-type (WT) tree by using massively parallel signature sequencing (MPSS). A total of 16,564,500 and 16,235,952 high quality reads were obtained for the WT and the mutant (MT), respectively. Interpretation of the MPSS signatures revealed that the total number of transcribed genes in the MT (31,468) was larger than in the WT (29,864), suggesting that newly initiated transcription occurs in the MT. Further comparison of the transcripts revealed that 2735 genes had more than twofold expression difference in the MT compared with the WT. In addition, we identified 110 citrus flowering-time genes homologous with known elements of flowering-time pathways through sequencing and bioinformatics analysis. These genes are highly conserved in citrus and other species, suggesting that the functions of the related proteins in controlling reproductive development may be conserved as well.

**Conclusion:**

Our results provide a foundation for comparative gene expression studies between WT and precocious trifoliate orange. Additionally, a number of candidate genes required for the early flowering process of precocious trifoliate orange were identified. These results provide new insight into the molecular processes regulating flowering time in citrus.

## Background

Flowering is one of the most important aspects of development in plants to ensure successful reproduction and eventual adaptation to surrounding environments. Plants have evolved mechanisms to integrate various environmental signals, including photoperiod and vernalization, to enable flowering under conditions that optimize seed production [1,2]. In recent years, molecular and genetic regulation of flower development has been extensively investigated in herbaceous plants, particularly in *Arabidopsis *[3-5]. A number of different pathways have been described in *Arabidopsis *that induce the floral transition, including vernalization, photoperiod, autonomous, and gibberellin (GA) pathways that form a regulatory network [6,7]. Genes involved in controlling the timing of floral transition have been identified through mutagenesis and analysis of natural variation. These four promotion pathways are integrated through the transcriptional regulation of two "flowering-time" genes, *FLOWERING LOCUS T *(*FT*) [8-10] and *SUPPRESSOR OF OVEREXPRESSION OF CONSTANS 1 *(*SOC1*), and two floral meristem identity genes, *LEAFY *(*LFY*) and *APETALA1 *(*AP1*) [10-13]. Among the integrators, *FT *and *SOC1 *have a more direct function in determining flowering time. They share the common upstream regulators *CONSTANS *(*CO*), a key component in the long day pathway, and *FLOWERING LOCUS C *(*FLC*), a flowering repressor integrating autonomous and vernalization pathways [10]. *FLC *suppresses flowering, at least in part, by repressing the expression of the floral activators *SOC1 *and *FT*, and it has been shown to bind directly to these activators [14,15]. *LFY *is a key player in the specification of floral meristem identity [16], and it dramatically increased the number of coflorescences in a *lfy *mutant due to the activity of *AP1 *[7]. Thus, crosstalk between pathways might explain how the multiple signals affecting flowering are coordinated, and differences in how pathways are integrated might underlie the diversity of plant flowering [3,6,17].

Regulation of flowering in woody perennials shows remarkable differences with respect to herbaceous species. Annual plants complete their life cycle in one year and initiate flowering only once, whereas most other fruit crops have a long juvenile period, during which no reproductive development occurs. After this period, however, flowering happens repeatedly. How perennial woody plants undergo a long silent juvenile stage and then repeated vegetative growth and flowering has not been extensively studied at the level of transcription [17].

Citrus is one of the most widespread fruit crops in the world, with great economic and health value [18]. It is among the most difficult plants to improve through traditional breeding approaches due to undesirable reproductive traits and characteristics [19]. These include degrees of sexual sterility and incompatibility, nucellar embryony, extended juvenility, and large plant size. To accelerate flowering time, constitutive expression of *LFY *or *AP1 *derived from *Arabidopsis *was used to dramatically reduce generation time in citranges [20]. In a similar approach, but with a citrus gene, it was shown that transgenic poncirus carrying the *CiFT *(an *FT *homolog) also exhibited early flowering, although this phenotype was accompanied by several pleiotropic effects [21]. In addition, the long juvenile phase (6 to 8 years) of most citrus genotypes impedes the rapid evaluation of transgenic trees modified to affect adult traits such as fruit quality (e.g., rind and flesh color, flavors, maturity dates, seediness, abscission, peelability, rag content, acid and sugar levels) and other traits associated with productive mature trees. Thus, understanding the molecular regulation of the flowering process is crucial for controlling fruit production in citrus.

In 1976, precocious trifoliate orange with a short juvenile phase, a spontaneous mutant (MT) derived from *Poncirus trifoliata *(L.) Raf, was found in Yichang, Hubei province, China. In contrast to the 6- to 8-year juvenile period of the wild-type (WT) trifoliate orange, almost all of the seedlings that germinated from the MT had only a 3-year juvenile period; 20% of the seedlings even flowered in the year after germination. The MT seedlings can flower two to three times per year, while the WT plant flowers only once per year. The MT and the WT had nearly the same morphological characteristics aside from flowering habit, and no DNA polymorphisms were detected between them. Consequently, the MT was speculated to be a direct variant of the WT, which was ideal for studying floral induction, inflorescence development, and the flowering molecular mechanism. Therefore, a transcriptional study including a cDNA macroarray in combination with suppression subtraction hybridization (SSH) was used to investigate gene expression changes in the MT, and a total of 368 differentially expressed genes were detected [22-25]. Interestingly, most of the 368 genes showed differential expression in the year after self-pruning, indicating that this period is a critical stage for the transcriptional regulation of the MT trait formation. Taken together, our previous research using SSH technology has provided important clues for understanding the formation of the mutation trait in precocious trifoliate orange; however, the transcriptional information from SSH, especially for genes expressed at low levels, is rather limited. Further analyses of gene expression during the early flowering process is needed.

Massively parallel signature sequencing technology (MPSS), like expressed sequence tags (ESTs) and serial analysis of gene expression (SAGE), is a sequence-based method that can be used for transcriptional profiling to measure gene expression. However, MPSS provides more thorough qualitative and quantitative descriptions of gene expression due to its tremendous depth [26-30]. This depth enables identification of a nearly complete inventory of transcripts in a given sample [31]. To date, the MPSS method has provided a rapid way to identify and profile differentially expressed genes in a variety of plants, mutants, and tissues, and at different stages of development [28,30-32]. In this study, a genome-wide gene expression study was carried out between WT and precocious trifoliate orange by using MPSS, and a total of 36,523 genes were analyzed. Of these genes, 2735 showed a twofold or greater expression difference between the MT and WT. The results demonstrated that some genes may be newly transcribed in the MT. Our results also identified a large number of genes previously not known to be involved in the early flowering development process. Interpretation of the data solidified links between new information herein and our previous fragmentary knowledge, and provided new insight into the molecular processes regulating flowering time in citrus.

## Results

### Flowering characterization of WT and precocious trifoliate orange

**Figure 1:**
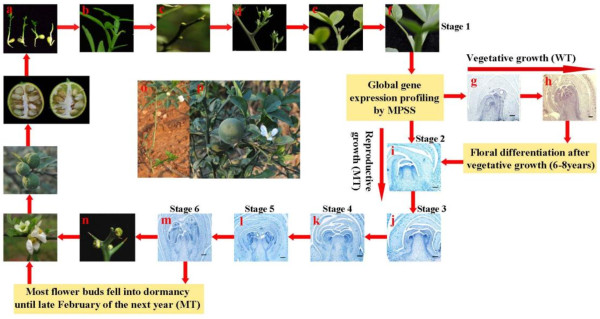
**Schematic diagram of developmental stages involved in the flowering of precocious trifoliate orange**. **a**, Seedling of precocious trifoliate orange (1 month). **b**, The shoot apical meristem of seedling begins self-pruning (October). **c**, The terminal bud and lateral buds of the juvenile trees became dormant after self-pruning (mid-October). **d**, The lateral buds develop into a spring shoot in the next year (late February). **e**, The spring shoot begins self-pruning (early April). **f**, After self-pruning (mid-April). **g, h**, The lateral bud becomes a leaf bud (from late April to mid-May). **i**, Sepal primordia arise (late April). **j**, Petal primordia arise (late April). **k**, Stamen primordia arise (early May). **i**, Pistil primordia arise (mid-May). **m, n**, Full-developed floral bud (late May). **o, p**, The early flower morphology of precocious trifoliate orange.

Self-pruning is a physiologic phenomenon in trifoliate orange in which shoots cease vegetative growth by automatically withering the shoot tip (0.5-1 cm, Figure 1b and 1e). Self-pruning is a necessary but insufficient condition for floral bud initiation. Until the late stage of self-pruning, the shoot apical meristem of juvenile trifoliate orange is in an undetermined state and floral primordia are not observed (October, Figure 1b). After self-pruning, the terminal bud and lateral buds of the juvenile trees enter dormancy until late February of the next year (Figure 1c). (In citrus, there are three flushes during the growing season. The spring flush is the most important one for growth and flower formation; these lateral buds develop into spring shoots in the next year.) A major characteristic of the MT is that its juvenile phase is shortened to 1 to 2 years, whereas the WT plant has a long juvenile period of 6 to 8 years. This "nonapparent" growth period is important for flower induction and the transition from the vegetative to the flowering stage of precocious trifoliate orange. Cytological observation revealed that the floral buds in the precocious mutant initiated differentiation immediately after self-pruning on spring shoots (Figure 1d-1f). Floral development hastened differentiation and produced the primordia of sepal, petal, stamen, and pistil sequentially. The whole flower bud integrates formation in one month, and then part of the flower bud population began to flower (Figure 1i-1m). However, most flower buds fell into dormancy until late February of the next year. For WT trifoliate orange, the spring shoots, which did not form floral buds, began to produce vegetative buds after self-pruning (Figure 1g and 1h).

### MPSS signature abundance and distribution

MPSS libraries were constructed using RNA extracted from the terminal bud and the five following buds of spring shoots (after self-pruning) for the MT and the WT. A total of 16,564,500 and 16,235,952 successful reads were produced for MT and WT, respectively (Table 1). The sequence sets were filtered to remove low quality reads containing ambiguous nucleotides and adaptor sequences in both libraries, resulting in 16,067,565 (97.00%) clean reads for MT and 15,701,789 (96.71%) clean reads for WT (clean reads are termed as "signature" hereafter). The signature sets were filtered to remove any signatures that were not 1) reliable, i.e., observed in only one sequencing run, and 2) not significant, i.e., never observed at or above 3 transcripts per million (TPM) in either library. Of the 16,067,565 signatures for MT, 34.2% (5,490,689) did not meet the reliability criterion (Table 1). Meanwhile, of the 15,701,789 signatures of the WT library, 33.0% (5,175,486) did not meet the reliability criterion. After filtering, 10,576,876 reliable signatures were observed in the MT library, with 10,527,303 reliable signatures in the WT library. The final set of reliable and significant signatures comprised 31,468 unigenes for the MT library and 29,864 unigenes for the WT library. When the data from the two libraries were combined, a total of 36,523 nonredundant unigenes were observed. Of these, 6,859 were not observed in the WT library and 5,055 were not present in the MT library (Table 1).

**Table 1:** Summary statistics of MPSS signatures in the precocious trifoliate orange (MT) and its wild-type (WT)

	MT library	WT library
Total reads	16,564,500	16,235,952
Total nucleotides (nt)	1,242,337,500	1,217,696,400
High-quality reads	16,067,565	15,701,789
High-quality reads (%)	97.00	96.71
Low quality reads	496,935	534,163
Low quality reads (%)	3	3.29
GC percentage of high-quality reads (%)	44.64	45.06
Reliable significant reads	10,576,876	10,527,303
Number of contigs	83,412	81,148
Number of singletons	46,046	43,119
Number of unigenes	31,468	29,864

**Figure 2:**
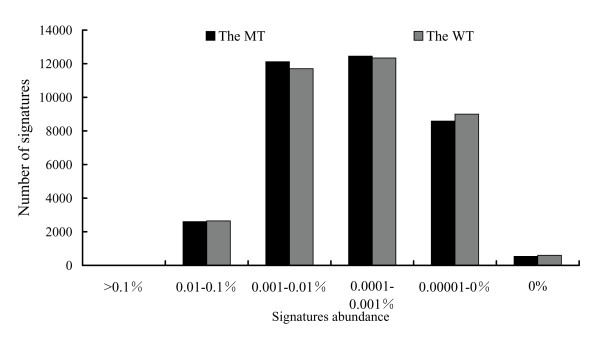
**The MPSS signature abundance distributions**. The abundance of each signature is calculated as a percentage of total signatures in the mutant (black column) and wild-type (gray column).

We found that MPSS was able to detect many transcripts expressed at low levels. Figure 2 shows the distribution of MPSS signatures at different abundance levels in TPM. The distribution of signature abundances across both libraries was generally quite similar. Three signatures in MT and four signatures in WT were expressed at a high abundance (more than 0.1%, or > 1000 TPM). While the number of signatures increased dramatically with a decrease in abundance, 74.88% of the total signatures in MT and 73.56% of the total signatures in WT had an abundance of less than 0.0001% (Figure 2). Moreover, of the signatures present in a given library, the vast majority, 93% of the WT library and 98% of the MT library, were below 0.001% abundance (< 100 TPM) (Figure 2). This illustrates the sensitivity of next generation sequencing technology in identifying transcripts with low expression. Of the total signatures, 1.6% of them were not found in the MT library and 1.7% of them were not found in the WT library (Figure 2).

### Differential expression of MPSS signatures between MT and WT plants

**Figure 3:**
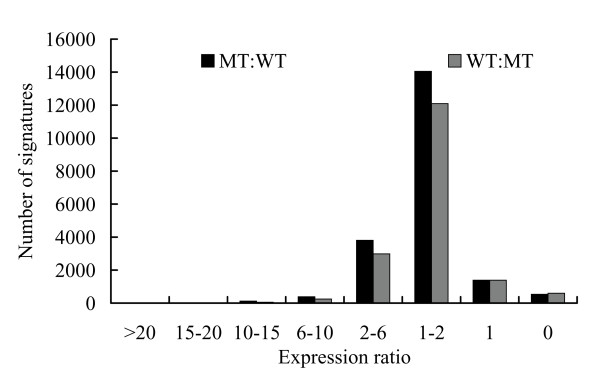
**Comparison of expression of the MPSS signatures between the mutant (black column) and wild-type (gray column)**. The expression ratios compare the abundance of each signature between the mutant and wild-type. Columns denote the number of signatures with an expression ratio within the stated range.

**Figure 4:**
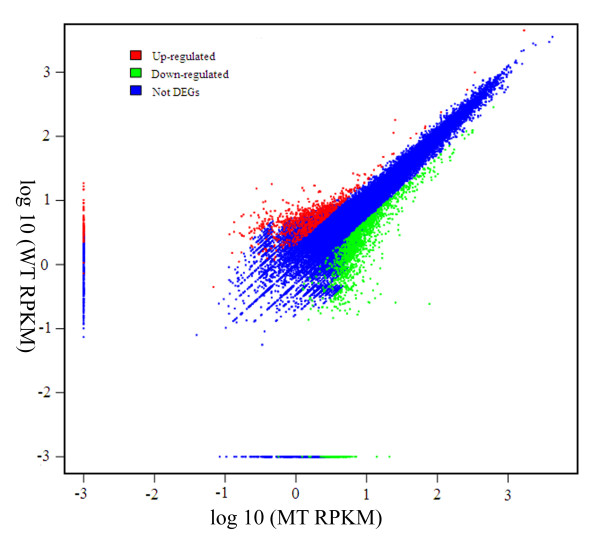
**Comparison of unigenes expression between the mutant and the wild-type**. The abundance of each gene was normalized as transcripts per million (TPM). The differentially expressed genes are shown in red and green, while blue indicates genes that were not differentially expressed genes (not DEGs) between the mutant and the wild-type.

The frequency of signatures was regarded as the relative expression level of each transcript in MT and WT libraries. Comparative analyses of the signature frequencies between MT and WT revealed that the expression ratio (MT/WT) varied greatly from 0.009 to 318. Only 3.6% signatures were species specific, in that they were found only in one library and were absent from the other (expression ratio = 0, Figure 3). Of the common signatures in both libraries, only 26.5% of all signatures (9670) showed a twofold or greater (ratio > 2 or < 0.5) expression difference between MT and WT, and were regarded as differentially expressed transcripts according to the criteria defined by Meyers et al. [33]. Of these, 19.9% had expression ratios between 2 and 5, and only a small percentage of signatures (3.6%) showed more than a fivefold difference in expression level between the two libraries (Figure 3). Signature frequency was also compared statistically between the two libraries using the Z-score method according to Kal et al. [34], which uses the p-value and a statistical significance value. The expression of 2,735 signatures was significantly different at p < 0.005, at the same time their expression ratios were greater than 2 or less than 0.5 (Figure 4). Among these, 1000 genes were down-regulated in the MT compared to the WT and 1735 genes were up-regulated. Of these 2735 signatures, 1855 (23%) were significant at p < 0.001.

### Length distributions and functional annotation of unigenes

The average length of unigenes was 674.41 and 665.35bp for the WT and MT library, respectively, which is shorter than those of *Arabidopsis *(1445bp) and soybean (1539bp) [35,36]. The median value for the length of the unigenes was 1020 and 1002bp for the WT and MT library, respectively, which is shorter than *Arabidopsis *(1459bp) and rice (1548bp) [37], but longer than poplar (990bp) [38]. The average length of the open reading frame was 718 and 711 bp, corresponding to an average polypeptide length of 239 and 237aa, respectively, which was also shorter than those of *Arabidopsis*, rice, and soybean, and longer than poplar and maize [39,40]. Additional File 1 provides the distributions of cDNA length and the CDS length from 31,468 and 29,864 nonredundant unigene sequences, respectively.

Our annotation method was based on sequence homology searches and the annotations that accompanied them. Its aim was to capture the most informative and complete annotation possible. Table 2 shows the hit numbers and percentages relative to those of the major public databases. These annotation statistics show all the unigenes annotated by the BLAST search against the public protein and nucleotide databases (SwissProt, KEGG, COG, and Nr) where the E-value threshold was set at 1E-5. Of the 31,468 MT and 29,864 WT unigenes, 23,835 and 23,076 unigenes had at least one hit within these databases, respectively (Table 2). The remaining unigenes (24.26% and 22.73%, respectively) that were not annotated likely comprised citrus-specific genes, as well as genes with homologs in other species whose corresponding biological functions have not yet been investigated. In addition, proteins with the highest ranks in the BLAST results were taken to decide the coding region sequences of the unigenes, and the coding region sequences were then translated into amino sequences with a standard codon table. Consequently, both the nucleotide sequences (5'-3') and amino sequences of the unigene coding region were acquired. Unigenes that cannot be aligned to any database were scanned by ESTScan [41] to get the nucleotide (5'-3') and amino sequences of the coding regions. A total of 25,318 genes (12,064 in sense and 12,254 in antisense) were identified in the MT library (Additional File 2), and 22,510 genes (11,681 in sense and 11,892 in antisense) were identified in the WT library (Additional File 3); a total of 7,150 (22.72%) for the MT and 6,354 (21.28%) for the WT library were not identified in sense or antisense. Transcription factors (TFs) are important regulators for activating or repressing the expression of coding or noncoding genes, through which they can further influence or control many biological processes [42]. Putative TF genes were identified by a BLAST search against rice, *Arabidopsis*, and *Citrus sinensis *TF genes downloaded from PlantTFDB (http://planttfdb.cbi.pku.edu.cn:9010/index.php) [43], identifying 569 putative TFs belonging to 60 TF families; there were 552 TFs in the WT library and 564 TFs in the MT library (Additional File 4). The *MADS *family was the most prevalent, followed by the *AP2/EREBP *and *WRKY *families. These results were slightly different from those of *Arabidopsis *[33] and rice [30], in which the *AP2/EREBP *family and zinc finger family were predominant, respectively. In addition, the *AP2/EREBP *family and *C2H2 *family were slightly differentially expressed between the genotypes (Additional File 4).

**Table 2:** Hit percentages against important public databases^a^

	36,523 Unigenes (All)		29,864 Unigenes (WT)		31,468 Unigenes (MT)	
**Database**	**Annotated (n)**	**%**	**Annotated (n)**	**%**	**Annotated (n)**	**%**

SwissProt	19,119	52.35	16,431	55.02	16,675	52.99
KEGG	12,724	34.84	11,005	36.85	11,279	35.84
Nr	26,938	73.76	22,042	73.81	23,770	75.54
COG	9,515	26.05	8,397	28.12	8,446	26.84
Total	26,993	73.91	23,076	77.27	23,835	75.74

^a^E value threshold is E^-5^.

### Functional classification of differential expression genes

**Figure 5:**
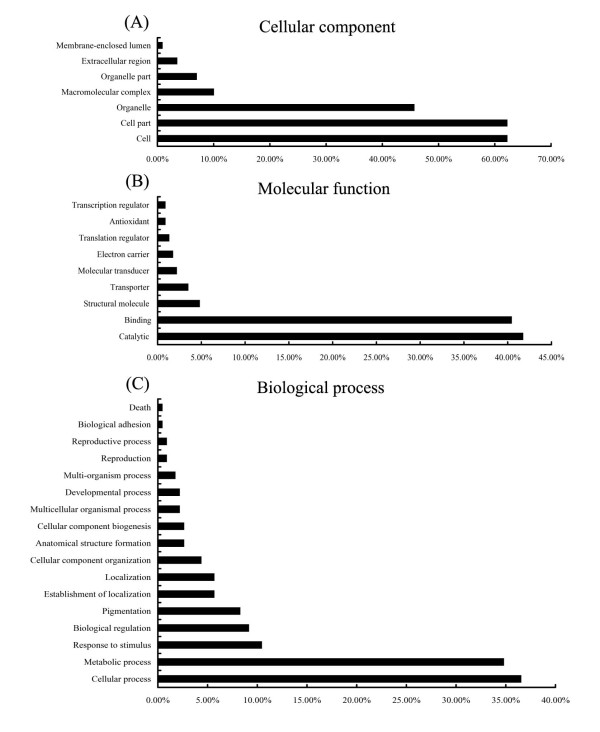
**Functional categorization of the genes with significant transcriptional changes between the mutant and the wild-type**. The genes were categorized based on Gene Ontology (GO) annotation and the proportion of each category is displayed based on **A**, cellular component, **B**, molecular function, or **C**, biological process.

As a result of the completed genomic sequencing of the plant *Arabidopsis*, the currently available expressed sequences have been invaluable in defining the correct components of the gene structure in this species [44]. To evaluate the potential functions of genes with significant transcriptional changes between the MT and WT, Gene Ontology (GO) categories were assigned to the significant 2735 genes based on the TAIR GO slim provided by blast2GO. The categorization of differential expression genes according to the cellular component, molecular function, and biological process is shown in Figure 5. With regard to cellular component, the analysis revealed a high percentage of cell parts and organelles. For categories based on molecular function, the genes were finally classified into eight categories, as shown in Figure 5B; the three most overrepresented GO terms were binding (nucleotide binding, protein binding, chromatin binding), catalytic, and transcription regulators. Differential expression genes were related to 17 biological processes, including cellular process, biological regulation, metabolic process, developmental process, response to stimulus, multicellular organismal process, and others (Figure 5C). The biological interpretation of the significant differential expression genes was further completed using KEGG pathway analyses (Additional File 5). A total of 200 different metabolic pathways were found in this study, with some being consistent with biological processes already revealed by GO analyses. The most represented pathways included metabolic pathways (168 enzymes represented), biosynthesis of secondary metabolites (100), plant-pathogen interaction (65), phenylpropanoid biosynthesis (45), spliceosome (38), and cell cycle (26). Of these, some were related to mutation trait formation based on previous knowledge, including biosynthesis of plant hormones, spliceosome, RNA degradation, ubiquitin-mediated proteolysis, and calcium signaling pathway. In addition, GO representations from this study were compared with a GO representation based on all the unigenes from sweet orange in the TIGR gene index database, and no significant differences were seen between the two groups.

### Identification of flowering-related genes

**Figure 6:**
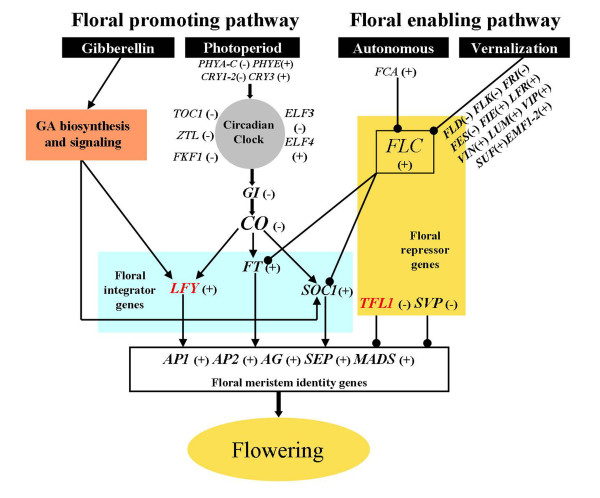
**Pathways regulating the floral transition in *Arabidopsis ***[68,69]. Long photoperiod and gibberellic acids (GAs) promote the floral transition by activating the floral pathway integrators. The enabling pathways regulate floral competence of the meristems by regulating floral repressor activity such as the *FLC*. Arrows indicate activation and short lines ending with a dot indicate repression. The data underlying the model and the corresponding homologs in citrus are presented in Table 1. A plus sign (+) indicates up-regulation and a minus sign (-) indicates down-regulation in precocious trifoliate orange. Genes in red type were not found by MPSS.

There are physical, chemical, and biological signals that lead to the onset of flowering. The four known pathways that respond to these signals have been characterized in *Arabidopsis *and some herbaceous model plants. In order to identify flowering-related differential expression genes in this study, putative functions of 36,523 nonredundant signatures were assessed by BLAST searches against the TAIR9, RAP-DB, and NCBI Nr protein datasets. We identified 110 citrus unigenes representing putative homologs to flowering-related genes. Some of these genes are required for the day length response and some encode regulatory proteins specifically involved in the control of flowering, while others encode components of light signal transduction pathways or are involved in circadian clock function. A representation of the relationships among these processes is shown in Figure 6 and the putative homologs of the key players in citrus are presented in Table 3. For example, we found some putative homologs for floral integrator or identity gene such as *FT*, *SOC1*, *FLC*, *CO*, and *AP1*.

**Table 3:** Citrus unigenes that share homology to flowering-time genes of other plants.

Gene ID	Gene Length	Reads (MT)	Reads (WT)	Accession number	Identity (%)	E-value	Protein function (species)
**Photoperiod pathway**							

U8650	1327	379	348	XM_002523031	728/1003 (72%)	5E-152	*PHYA (Ricinus communis)*
U20394	1643	233	275	XM_002512550	1175/1500 (78%)	0	*PHYA (Ricinus communis)*
U1862	4042	2219	2668	XM_002519184	2759/3356 (82%)	0	*PHYB (Ricinus communis)*
U11402	4270	748	829	EU436651	1638/2059 (79%)	0	*PHYC (Vitis vinifera)*
U29320	871	16	24	EU436656	541/690 (78%)	1E-163	*PHYE (Vitis riparia)*
U2208	3284	710	700	U39787	2162/2958 (73%)	0	*PHYE (Ipomoea nil)*
U8176	960	1809	1231	NM_112521	329/460 (71%)	4E-57	*PAP1 (Arabidopsis thaliana)*
U13637	1705	118	146	XM_002275300	1396/1706 (81%)	0	*PIE1 (Vitis vinifera)*
U10186	1611	49	92	XM_002275300	1052/1475 (71%)	0	*PIE1 (Vitis vinifera)*
U2658	3452	637	670	XM_002275300	2232/2909 (76%)	0	*PIE1 (Vitis vinifera)*
U12327	1159	447	554	NM_179665	59/75 (78%)	1E-06	*PIL5 (Arabidopsis thaliana)*
U21857	3019	1115	1080	NM_102365	1678/2388 (70%)	0	*PFT1 (Arabidopsis thaliana)*
U8820	1615	184	194	NM_119618	220/271 (81%)	5E-65	*CIB1 (Arabidopsis thaliana)*
U11824	2226	89	148	NM_120506	463/579 (79%)	1E-143	*ELF6 (Arabidopsis thaliana)*
U9378	598	14	16	NM_148863	212/268 (79%)	2E-57	*REF6 *(*Arabidopsis thaliana*)
U1219	3191	300	297	NM_148863	287/371 (77%)	6E-74	*REF6 (Arabidopsis thaliana)*
U9118	2795	3825	4339	FJ882041	1486/1938 (76%)	0	*CRY2 (Fragaria vesca)*
U1555	3639	439	401	NM_122394	359/463 (77%)	3E-97	*CRY3 (Arabidopsis thaliana)*
U9204	2765	1785	2109	NM_116961	1364/1731 (78%)	0	*CRY1 (Arabidopsis thaliana)*
U12873	3154	940	911	XM_002268467	2102/2575 (81%)	0	*FHY3 (Vitis vinifera)*
U20135	629	33	30	AY830926	82/105 (78%)	2E-27	*ELF4 (Pisum sativum)*
U31613	682	7	20	AY830926	153/213 (71%)	4E-24	*ELF4 (Pisum sativum)*
U18048	2019	507	568	NM_106379	352/491 (71%)	3E-62	*EFS (Arabidopsis thaliana)*
U9915	867	372	363	EU916963	475/636 (74%)	4E-113	*ELF4 (Citrus sinensis)*
U861	501	103	87	EU916963	380/461 (82%)	5E-122	*ELF4 (Citrus sinensis)*
U5852	687	497	341	EU916963	661/669 (98%)	0	*ELF4 (Citrus sinensis)*

**Circadian clock**							

U10400	2627	907	1052	DQ371901	1516/1849 (81%)	0	*ZTL2 (Glycine max)*
U17912	1648	1194	1240	AY611028	803/1067 (75%)	0	*TOC1 (Castanea sativa)*
U12636	870	81	108	AY611028	134/162 (82%)	1E-37	*TOC1 (Castanea sativa)*
U10936	3030	753	1022	NM_128153	94/109 (86%)	1E-25	*ELF3 (Arabidopsis thaliana)*
U10110	3033	812	934	AY371292	285/380 (75%)	7E-60	*ELF3 (Mesembryanthemum)*
U33021	293	12	17	NM_102715	84/117 (71%)	1E-06	*RKF1 (Arabidopsis thaliana)*
U10089	381	17	14	NM_102715	241/351 (68%)	4E-26	*RKF1 (Arabidopsis thaliana)*
U12503	2363	229	258	NM_130368	726/1031 (70%)	3E-127	*RKF3 (Arabidopsis thaliana)*
U9215	3251	2563	2990	XM_002524295	2328/2901 (80%)	0	*GI (Ricinus communis)*
U7045	559	765	891	XM_002524295	463/559 (82%)	5E-161	*GI (Ricinus communis)*
U9165	2436	716	860	NM_105475	1303/1747 (74%)	0	*FKF1 (Arabidopsis thaliana)*

**Vernalization pathway**							

U9585	1466	67	133	NM_111874	1079/1457 (74%)	0	*FLD (Arabidopsis thaliana)*
U22618	1361	200	211	NM_111874	367/527 (69%)	3E-54	*FLD (Arabidopsis thaliana)*
U23858	458	5	2	NM_111874	141/185 (76%)	1E-28	*FLD (Arabidopsis thaliana)*
U4972	283	72	95	NM_111333	217/281 (77%)	8E-53	*FLK (Arabidopsis thaliana)*
U2940	947	298	402	NM_111333	143/171 (83%)	2E-42	*FLK (Arabidopsis thaliana)*
U8380	1094	588	690	NM_111333	531/714 (74%)	3E-122	*FLK (Arabidopsis thaliana)*
U15692	502	860	282	EU497679	327/327 (100%)	2E-165	*FLC9 (Poncirus trifoliata)*
U12215	691	377	180	EU605888	668/671 (99%)	0	*FLC7 (Poncirus trifoliata)*
U11512	2236	258	293	XM_002511008	453/635 (71%)	2E-83	*FRI (Ricinus communis)*
U2597	2060	637	638	XM_002524905	1369/1728 (79%)	0	*FRI (Ricinus communis)*
U11002	1954	405	491	XM_002529001	289/452 (63%)	8E-51	*FRI (Ricinus communis)*
U767	2311	257	227	XM_002266277	289/452 (63%)	8E-51	*FRI (Ricinus communis)*
U17929	1689	828	767	NM_113199	926/1232 (75%)	0	*LFR (Arabidopsis thaliana)*
U14324	3620	950	928	XM_002520611	621/839 (74%)	2E-144	*LUM (Ricinus communis)*
U12966	1832	396	399	EU884426	937/1258 (74%)	0	*VRN2-1 (Malus × domestica)*
U8773	1681	1784	1643	EF064791	81/111 (72%)	9E-05	*VIN3-1 (Arabidopsis thaliana)*
U17262	1894	191	161	EF064791	218/294 (74%)	4E-41	*VIN3-1 (Arabidopsis thaliana)*
U11331	1533	813	846	EF064792	665/963 (69%)	1E-97	*VIN3-2 (Arabidopsis thaliana)*
U17602	1306	951	885	NM_119129	713/967 (73%)	5E-171	*VIP3 (Arabidopsis thaliana)*
U23612	1156	148	117	NM_112785	38/47 (80%)	1.4	*VRN1 (Arabidopsis thaliana)*
U8880	1600	512	515	NM_112785	284/384 (73%)	1E-59	*VRN1 (Arabidopsis thaliana)*
U7754	1102	273	191	NM_112785	127/183 (69%)	6E-11	*VRN1 (Arabidopsis thaliana)*
U9616	1943	550	469	NM_102836	748/1086 (68%)	5E-110	*SUF4 (Arabidopsis thaliana)*
U9937	953	38	41	GQ177180	86/120 (71%)	1E-06	*FES1 (Arabidopsis thaliana)*
U34190	1385	121	129	NM_179890	176/259 (67%)	2E-13	*FES1 (Arabidopsis thaliana)*
U12018	1939	288	248	NM_121191	63/81 (77%)	7E-07	*EMF1 (Arabidopsis thaliana)*
U11876	2468	769	751	XM_002281643	1519/2012 (75%)	0	*EMF2 (Vitis vinifera)*
U2303	1580	677	580	NM_112965	784/1037 (75%)	0	*FIE (Arabidopsis thaliana)*

**Autonomous pathway**							

U15708	416	21	23	XM_002519206	146/185 (78%)	4E-33	*FCA (Ricinus communis)*
U13760	1112	593	591	XM_002519206	146/185 (78%)	4E-33	*FCA (Ricinus communis)*
U9139	2236	883	868	AK229352	75/97 (77%)	2E-10	*FCA (Arabidopsis thaliana)*
U32661	983	8	8	AK229352	75/97 (77%)	2E-10	*FCA (Arabidopsis thaliana)*
U20071	955	126	82	AK229352	75/97 (77%)	2E-10	*FCA (Arabidopsis thaliana)*

**Floral pathway integrator**							

U19004	346	6	3	EU032531	167/170 (98%)	5E-76	*SOC1-1 (Citrus sinensis)*
U3347	353	22	8	EU032532	343/353 (97%)	4E-166	*SOC1- 2 (Citrus sinensis)*
U1570	283	8	6	EU032532	244/281 (86%)	2E-98	*SOC1-2 (Citrus sinensis)*
U27861	691	43	12	AB301935	672/695 (96%)	0	*CiFT (Citrus unshiu)*
U27120	303	12	0	AY338974	298/303 (98%)	1E-145	*AP1 (Citrus sinensis)*
U17717	1389	1066	1165	DQ371898	340/433 (78%)	5E-96	*CO2 (Glycine max)*
U2713	1871	799	962	NM_001125127	279/375 (74%)	9E-56	*CO9 (Arabidopsis thaliana)*
U17161	836	1317	1623	FJ719767	538/716 (75%)	4E-132	*CO (Mangifera indica)*
U8846	1586	2570	1905	GQ864262	250/360 (69%)	2E-32	*CO8 (Glycine max)*
U36503	1822	849	875	GQ864265	114/140 (81%)	4E-28	*CO (Glycine max)*
U9364	1051	128	123	GQ864265	92/129 (71%)	4E-07	*CO (Glycine max)*
U2300	1394	589	840	GQ864263	116/147 (78%)	6E-25	*CO9 (Glycine max)*

**Other flowering genes**							

U5505	430	23	13	U78949	331/396 (83%)	2E-114	*MADS3 (Malus domestica)*
U13903	870	147	137	DQ500880	470/617 (76%)	4E-126	*MADS3 (Populus tomentosa)*
U1579	1210	152	93	XM_002301057	526/669 (78%)	2E-156	*MADS9 (Populus tomentosa)*
U6983	545	908	706	AB218613	543/545 (99%)	0	*CitMADS6 (Citrus unshiu)*
U34186	773	14	35	AB218614	440/551 (79%)	2E-136	*CitMADS8 (Citrus unshiu)*
U24167	336	9	5	AB218611	286/336 (85%)	0	*CitMADS3 (Citrus unshiu)*
U12163	996	276	85	AB218612	677/686 (98%)	0	*CitMADS5 (Citrus unshiu)*
U217	490	22	31	NM_001084836	78/96 (81%)	1E-15	*AGL16 (Arabidopsis thaliana)*
U9295	1938	2427	2378	NM_101733	308/390 (78%)	9E-88	*AGL65 (Arabidopsis thaliana)*
U8625	2045	400	316	NM_105623	343/487 (70%)	1E-55	*AGL94 (Arabidopsis thaliana)*
U20726	1826	257	166	XM_002326128	450/604 (74%)	7E-102	*AP2 (Populus trichocarpa)*
U12861	1107	443	679	FJ809943	818/820 (99%)	0	*AP2 (Poncirus trifoliata)*
U23896	275	11	3	AY256859	210/277 (75%)	3E-45	*AP1 (Vitis vinifera)*
U24321	344	14	5	GU357461	218/335 (65%)	2E-11	*SEP1 (Euptelea pleiosperma)*
U4356	1096	319	190	AB218614	627/636 (98%)	0	*CitMADS8 (Citrus unshiu)*
U3656	2411	1156	1400	NM_180137	534/715 (74%)	5E-130	*SPL1 (Arabidopsis thaliana)*
U12668	2514	1897	1917	AJ011628.1	437/609 (71%)	0	*SPL1(Arabidopsis thaliana)*
U9906	4081	1684	1828	NM_101951	1803/2671 (67%)	0	*SPL14 (Arabidopsis thaliana)*
U3220	3148	854	790	NM_180519	1221/1790 (68%)	2E-168	*SPL7 (Arabidopsis thaliana)*
U11760	1435	168	143	HM018601	335/469 (71%)	8E-62	*SPL10 (Vitis vinifera)*
U17573	1158	162	161	XM_002517836	319/400 (79%)	5E-95	*SPL (Ricinus communis)*
U31888	713	50	41	FJ502237	595/639 (93%)	0	*SPL9 (Poncirus trifoliata)*
U23754	732	15	11	FJ502238	563/576 (97%)	0	*SPL13 (Poncirus trifoliata)*
U12575	1329	71	81	FJ502238	1272/1326 (95%)	0	*SPL13 (Poncirus trifoliata)*
U8652	1325	1020	1130	NM_180137	734/1115 (65%)	9E-86	*SPL1 (Arabidopsis thaliana)*
U9909	2323	99	162	NM_105584	176/240 (73%)	1E-30	*SPL6 (Arabidopsis thaliana)*
U12495	1047	460	397	FJ373211	428/435 (98%)	0	*SVP (Poncirus trifoliata)*
U10522	1710	454	450	AB290727	719/1066 (67%)	8E-88	*TFL2 (Malus × domestica)*

Most genes of the *Arabidopsis *photoperiodic pathway were found in 36,523 nonredundant unigenes. We found several genes encoding putative photoreceptor apoproteins including *Phytochrome A-C*, *Cryptochrome 1-3*, *ZEITLUPE *(*ZTL*), and *FLAVIN BINDING KELCH REPEAT F-BOX 1 *(*FKF1*) (Table 3). In addition to genes of the photoperiodic pathway, homologs for both known sequences belonging to light quality pathways, *PHYTOCHROME AND FLOWERING TIME 1 *(*PFT1*) and *RELATIVE OF EARLY FLOWERING 6 *(*REF6*), were found. Of the central circadian clock genes, homologs of *LATE ELONGATED HYPOCOTYL (LHY) *and *TIMING OF CAB 1 (TOC1) *were also present in the two libraries (Table 3), but CCA1 was lacking. Furthermore, we found seven citrus unigenes that showed significant similarity to the *Arabidopsis CO*; these sequences were initially organized in six clusters. Among the regulators of *CO *transcription and protein stability, *GIGANTEA *(*GI*) was identified in the two libraries (Table 3).

For the vernalization pathway, we were not able to find *MAF*-like sequences (*MAF1*-*MAF5*: *MADS AFFECTING FLOWERING1-5*) from our libraries by BLAST searches (Table 3). However, we identified several homolog genes belonging to the *FRIGIDA *(*FRI*) complex as well as other regulatory complexes (*FRIGIDA-ESSENTIAL1*, *SUPPRESSOR OF FRIGIDA4*, and *MINIDEPENDENS*) involved in regulating the expression of *FLC*. Also putative members of *FLC *repressing the PRC complex were present in the two libraries. These included putative *VERNALIZATION INSENSITIVE 3 *(*VIN3*), *FERTILIZATION INDEPENDENT ENDOSPERM *(*FIE*), *EMBRYONIC FLOWER 1 *(*EMF1*), *VERNALIZATION 1 *(*VRN1*), and *LIKE HETEROCHROMATIN PROTEIN 1 *(*LHP1*). In addition, a putative homolog for *FLOWERING LOCUS D *(*FLD*) sequence was identified in this study (Table 3); *FLD *plays a key role in regulating the reproductive competence of the shoot and results in different developmental phase transitions in *Arabidopsis*. Lesions in *FLD *result in hyperacetylation of histones in *FLC *chromatin, up-regulation of *FLC *expression, and extremely delayed flowering [45].

In addition to the photoperiod and the vernalization pathways, we searched candidate genes for autonomous and GA pathways. Several sequences corresponding to *Arabidopsis *genes from both pathways were identified, suggesting the presence of these pathways in citrus. Among these genes we found homologs for *Arabidopsis FVE *and *SVP*, which have been shown to control flowering in a specific thermosensory pathway. Moreover, some additional flowering-time regulators that have not been placed in any specific pathway were identified, such as the MADS transcription factor family genes, *SQUAMOSA PROMOTER BINDING PROTEIN *family genes, *AGAMOUS *family genes, and *SEPALLATA *family genes (Table 3).

### Differential expression of flowering genes between the MT and WT plants

We compared the expression of selected flowering-time genes corresponding to each flowering pathway in the MT library and the WT library in order to explore the role of different pathways. Almost all flowering-related genes were differentially expressed between the genotypes; only a few of the genes were specifically expressed (Table 3). Of these, *AP1 *was not observed in the WT library, although a citrus *AP1 *had previously been identified [46]. The *AP1 *specifies flower meristem identity and is also required for normal development of sepals and petals. In the MT library, most flowering-promoting genes had slightly up-regulated expression compared with the WT library, such as *FLD*, *PFT1*, and *SEP1*. Floral integrator genes (*FT*, *SOC1*, and *FLC*) were clearly up-regulated in the MT library (Figure 6). On the other hand, most repressors of flowering genes were slightly down-regulated in the WT library compared with the MT library (Figure 6).

### Verification of the genes related to MT trait formation

**Figure 7:**
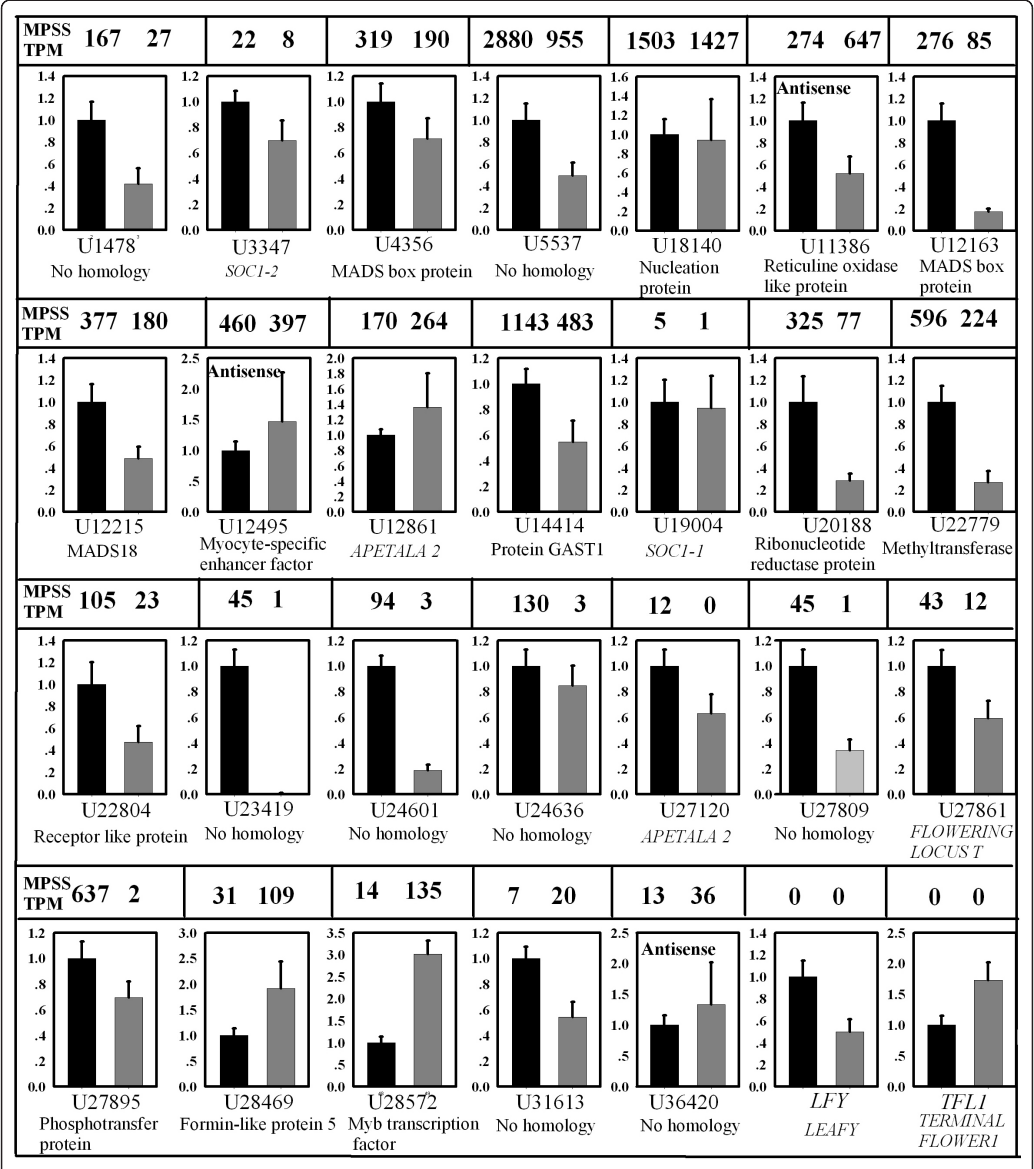
**Real-time quantitative RT-PCR confirmation of the differentially expressed genes between the mutant (black columns) and the wild-type (gray columns)**. The transcript abundance from MPSS data is shown above each gene; TPM, transcripts per million. Relative transcript levels are calculated by real-time PCR with *β-actin *as the standard. Data are means ± SE of three separate measurements.

**Figure 8:**
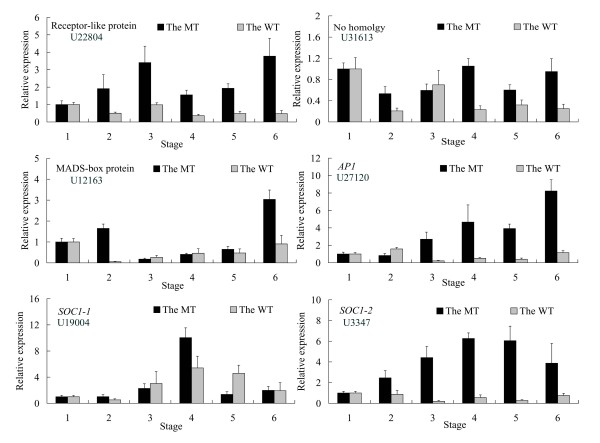
**Transcript level of six selected genes at different stages of spring shoot development in the mutant (black columns) and wild-type (grey columns)**. Stage 1: After self-pruning (undetermined lateral bud); Stage 2: Sepal primordia arise; Stage 3: Petal primordia arise; Stage 4: Stamen primordia arise; Stage 5: Pistil primordia arise; Stage 6: Fully developed floral bud. Relative transcript levels are calculated by real-time PCR with *Actin *as a standard. Data are means ± SE of three separate measurements.

Transcriptional regulation revealed by MPSS data was confirmed in a biologically independent experiment using quantitative reverse transcription polymerase chain reaction (RT-PCR). A total of 30 genes were chosen to design gene-specific primers (Additional File 6); these genes included 26 significantly differentially expressed genes, two genes of no differential expression, and two genes encoding proteins previously reported to be associated with, or involved in, developmental processes in other species. The transcript abundance patterns of the MT and WT were compared with MPSS data. Results showed that for 28 of the 30 genes, real-time PCR revealed the same expression tendency as the MPSS data, despite some quantitative differences in expression level. Figure 7 showed differential expression levels for 28 genes (20 for induced, three for repressed, three for antisense, and two previously reported flowering-related genes) between MT and WT. For example, the floral integrator gene *FT *was up-regulated 1.7 times in MT compared with WT as analyzed by real-time PCR, consistent with MPSS data showing that the gene's expression in the MT was threefold higher than in the WT. Furthermore, the expression profile of six genes, including some citrus putative homologs for floral integrator or identity genes (*SOC1 *and *AP1*), and three other newly detected genes with significant transcriptional changes were analyzed at six stages during flowering development between the MT and WT (Figure 8). As expected, the expression of these genes was correlated with floral induction, inflorescence development, and flowering of precocious trifoliate orange. It is notable that the expression levels of induced genes were up-regulated in the spring shoot apex samples of WT and MT genotypes before the floral initiation had occurred and repressed genes were down-regulated (Figure 8).

## Discussion

We explored expression patterns at a specific stage of flowering development in an early flowering trifoliate orange mutant by MPSS. MPSS and ESTs can be used for quantitative measurements of gene expression when combined with genomic sequencing [28,29]. Both approaches describe similar patterns of transcript abundance, although there are some clear differences that are perhaps associated with the methods themselves. Compared with EST technologies, MPSS involves deep transcript sampling and sequencing, usually 1-2 million transcripts per library, of a cDNA library on microbeads [29]. In principle, the MPSS data should provide a more thorough and quantitative representation of the absolute transcript population in terms of representation and relative abundance than EST data [28,30,47,48]. When compared with microarray technology that requires previous knowledge of genes, the limitations to detecting unknown genes are not encountered in MPSS [48,49]. On the other hand, compared to microarrays, a high-throughput sequencing approach is technologically more complex, yet much simpler statistically and methodologically [50]. The technology has been shown to provide comprehensive coverage and a sensitive measure of gene expression [51,52]. MPSS profiling has been used in studies to address various biological questions, including a whole-genome transcript analysis in *Arabidopsis *[53] and in human [51], and transcript expression profiling of hybrid and inbred parents in oyster [54]. From the results of this study, it is apparent that MPSS analyses not only highlight some genes and biological processes already revealed by our previous macroarray data [23,24], but also reveal a large number of genes that are possibly involved in the formation of the early flowering trait. The data consistency from multiple approaches ensures that the MPSS data produced in this study are reliable. In addition, the approximately 3.6 × 10^8 ^base pairs of data produced here represent a substantial sequence resource and will contribute to genomic data available for citrus.

To understand the transcriptome profile and to isolate flowering-related genes during flowering development in citrus, genome-wide gene expression profiling was compared between WT and precocious trifoliate orange by using MPSS. The results revealed 2735 differentially expressed genes that were induced or repressed more than twofold in the MT at the 0.05% significance level. We identified a large number of newly discovered, intriguing unigenes of transcription and post-transcription factors in these differentially expressed genes, indicating that these genes may be key regulators that control flowering development by activating or repressing numerous genes (Figure 7). Additionally, a number of putative homologs of genes for flowering time and floral organ identity were also found. To obtain additional insights into the functions of the differentially expressed genes, we examined the GO categorization for the most similar *Arabidopsis *homologs of each gene using functions within the TAIR website. Functional category analyses revealed that a number of important pathways may work collaboratively in shaping the early flowering trait in the MT. Genes encoding proteins categorized as including binding (nucleotide binding, protein binding, and chromatin binding), biological regulation, and developmental processes were enriched among those genes differentially expressed between the WT and the MT. We found a large proportion of "no homology" and similarity to unknown proteins sequences in the GenBank database. The underlying genetics of flower induction and floral organ formation may differ between woody perennials and herbaceous model species. Some of these genes may play important roles from the vegetative phase to floral development in MT. In addition, a high proportion of antisense transcripts, i.e., 12,254 of 25,318 genes in MT and 11,892 of 22,510 genes in WT, were observed in our study. These results suggested that the early flowering trait could be regulated on transcriptional and posttranscriptional levels. In contrast, the MPSS data provided much more information for the regulation of these activities on the transcriptional level. A notable result is that a considerable percentage of the genes, 9% of the total annotated genes, were involved in the regulation of biological processes or transcription (Figure 5). The induction of regulatory genes of transcription correlates well with the increased overall transcription in the MT. The total number of transcribed genes in the MT was 31,468, more than in the WT (29,864), suggesting that newly initiated transcription occurs in the MT. Moreover, analyses of the genes with transcription changes greater than twofold showed that up-regulated genes constituted 63.4% of the total changed genes. This suggests that these genes are related to uncharacterized mechanisms in the perception of signals and the initiation of flowering development.

For comprehensive identification of candidate genes in the citrus flowering pathways, we were able to identify 110 homolog genes among about 36,523 unigene sequences, representing putative citrus homologs to flowering-time genes (Table 3, Additional file 7). Sequences found in citrus corresponded to all known *Arabidopsis *flowering-time pathways, suggesting that all of these genetic pathways may be present in citrus. Exogenous GAs have been shown to inhibit flower bud formation not only in citrus [55] but also in apples [56], pears [57], and cherries and peaches [58]. This hormone is thus believed to strongly participate in regulating flower bud formation. Many GA biosynthetic and catabolism-related genes were also found in this study; however, we did not find clear differences in the expression of GA biosynthetic and catabolism-related genes in the spring shoot apex samples of WT and MT genotypes before floral initiation occurred. So these data do not support the role of the GA pathway as the regulator of early flowering process in the MT, indicating that GA signal may be regulated during flowering development of the two gene types rather than only in the MT.

In *Arabidopsis*, the floral induction signals from these four major flowering pathways (photoperiod, autonomous, vernalization, and GA-induced pathways) are transmitted to three flowering pathway integrators, *FT*, *SOC1*, and *LFY*. When *SOC1 *is induced at the shoot apex, *SOC1 *together with *AGL24 *directly activates *LFY*. *AP1*, activated mainly by *FT*, is also necessary to establish and maintain flower meristem identity [59]. When *LFY *and *AP1 *are established, flower development occurs at the anlagen of the shoot apical meristem according to the ABC model. During early flower development, *AP1 *activates the A function and represses three redundantly functioning flowering time genes, *SOC1*, *AGL24*, and *SVP *to prevent floral reversion. During late flower development, such repression is also necessary to activate *SEPALATA3 *(*SEP3*) which is a coactivator of B and C function genes with *LFY*; otherwise *SEP3 *is suppressed by *SOC1*, *AGL24*, and *SVP *[59]. We have found citrus homologs for all of these genes except *LFY *and *AGL24 *in this study; one of the flowering-promoting genes was up-regulated in the MT library compared with the WT library (Table 3). Nevertheless, it is clear that the citrus genome contains orthologs to *LFY*. Accordingly, overexpressing the citrus *LFY *DNA sequence dramatically induced early flowering in transgenic *Arabidopsis *[46]. Expression analysis of *LFY *by real-time RT-PCR showed that a transcript level of the gene was significantly up-regulated in the MT before the floral initiation had occurred (Figure 7). Therefore, we speculated that *LFY *is necessary to induce early flowering of precocious trifoliate orange. Citrus homologs for *SOC1*, *FT*, and *AP1 *were isolated previously and evaluated for their function in citrus or *Arabidopsis *[21,46,60,61]. They play important roles from the vegetative phase to floral development in citrus. *FT *is a member of a small gene family in *Arabidopsis *that also contains *TERMINAL FLOWER 1 *(*TFL1*). Although *TFL1 *has been functionally characterized in citrus, we were not able to identify *TFL1 *sequences in this study. Our previous work also revealed that a large portion of the promotion of flowering by *FT *and *TFL1 *was achieved through down-regulation of *TFL1 *levels and up-regulation of *FT *levels in the MT [24]. One possible explanation for why these previously reported flowering-related genes were not discovered in our library is that their mRNA transcript levels were too low to be measured by sequencing.

In *Arabidopsis*, *CO *encodes a zinc finger protein that acts as a floral activator and mediates the photoperiod pathway, whereas FLC encodes a MADS box protein that acts as a floral repressor and mediates the autonomous and vernalization pathways. *CO *and *FLC *regulate the expression of downstream genes, *FT*, *SOC1*, and *LFY *(Amasino 2010; Parcy 2005; Simpson and Dean 2002). We have identified seven unigenes showing significant (E-value lower than E-10) similarity to the *Arabidopsis CO *in two libraries. However, only a few of the *CO*-like genes were differentially expressed between the genotypes. Valverde et al. (2004) showed that the CO protein is ubiquitinated and then degraded by a protein complex called the proteasome, and that this process is regulated [62]. The autonomous and vernalization pathways promote flowering by repressing *FLC *expression and many genes involved in the vernalization and autonomous pathways control the epigenetic status of the *FLC *chromatin [14]. Although *FLC *homologous MADS box genes have been recently found from several eudicot lineages by phylogenetic analysis [63], *FLC *homologous genes have not been identified in any woody plants except citrus. Previous work in this mutant showed that specific splice variants of *FLC *were associated with transition from juvenile to mature trees, and four alternatively spliced transcripts of *FLC *were isolated [22]. In this study, not only were alternatively spliced transcripts of putative *FLC *homolog found, but *FRI *homologs could also be found in the MT library and the WT library. *FLC *was up-regulated in the MT library compared with the WT library, and the *FRI *expression pattern was contrary to that of *FLC*. Our previous work provided evidence that the expression profile of *FLC *was up-regulated during the winter, followed by a decrease in the spring and summer. This kind of cycling differs from the pattern observed in *Arabidopsis *[22]. A possible hypothesis suggests that due to the alternative splicing of *PtFLC *in citrus, which exerts its function in certain transcript form in the particular development stage, the total expression level of *PtFLC *was dispersed. Additionally, some sequences were found within the two libraries that would code for the other elements of the vernalization pathway: *VRN1*, *VRN2*, and *VRN3 *[64] or for the *VIP3*. *VRN2 *has a repressible role over the expression of *FLC *and codes for a protein with homology to PcG proteins [65]. These results indicate that the vernalization pathway may be present in citrus. Meanwhile, these data also suggested that these genes may play a critical role in the early flowering process of precocious trifoliate orange.

## Conclusions

In this study, we used the MPSS method to monitor global transcriptional changes in the MT compared with the WT, and identified 2735 differentially expressed genes that were induced or repressed more than twofold in the MT at 0.05% significance level. MPSS data analysis uncovered a large number of genes that were not previously known to be involved in formation of the mutation trait. A number of new genes possibly related to flowering time were found in this study. In addition, we explored 110 putative components for the genetic flowering pathways in citrus by identifying homologs of *Arabidopsis *flowering-time genes. The expression of selected flowering-time genes corresponding to each flowering pathway were compared in the MT library and the WT library, most flowering-promoting genes had up-regulated expression in the MT library, while most repressors of flowering genes had down-regulated expression in the MT library. These data also indicate that all known genetic flowering pathways may be present in citrus. The function of these elements can now be tested in heterologous systems, such as *Arabidopsis*, via transgenic approaches. We believe our results will be a valuable source for future research on the control of flowering and of biennial fruit-bearing patterns in citrus.

## Methods

### Plant material and RNA preparation

Wild-type and precocious trifoliate orange trifoliate orange samples were collected from the experimental fields of the National Citrus Breeding Center at Huazhong Agricultural University. The seeds of WT and precocious trifoliate orange trifoliate orange were planted in 20-cm pots containing a potting mix of a commercial medium and perlite at a ratio of 3:1. The juvenile potted seedlings were then transplanted and grown under field conditions. These juvenile trees were watered regularly with a nutrient solution. The terminal bud and the five following buds (the major node position for flower formation) from spring flushes of these MT and WT trees were collected after self-pruning, sepal primordia arise, petal primordia arise, stamen primordia arise, pistil primordia arise, and full-developed floral bud, respectively. All materials were collected from three individual plants and immediately frozen in liquid nitrogen and stored at -80°C until analyzed.

Total RNA was extracted according to a previous protocol [23]. A 1.2% agarose gel, stained with ethidium bromide, was run to preliminarily indicate the integrity of the RNA. All RNA samples were quantified and examined for protein contamination (A_260/280_) and reagent contamination (A_260/230_) by a Nanodrop ND 1000 spectrophotometer. In addition, the RIN (RNA integrity number) determined by the Agilent Technologies 2100 Bioanalyzer was greater than 8.5 for all samples.

### Massively parallel signature sequencing

The materials used for MPSS analyses were the terminal bud and the five following buds from MT and WT spring shoots after self-pruning, which occurs in mid-April (our previous results indicated that self-pruning is the critical stage for floral differentiation). Twenty micrograms of total RNA were sent to Beijing Genomics Institute (Shenzhen, China) where the libraries were produced and sequenced using Illumina's Genome Analyzer (Solexa). The MPSS was carried out essentially as in previous studies [29,66], with modifications from Long SAGE [67]. Briefly, cDNA with polyA/T tail was prepared and digested with *Dpn*II restriction enzyme. An adaptor with an *Mme*I recognition site was ligated to the 50-end, followed by *Mme*I digestion that cut 21-22 bases downstream. This 21-22 base signature from each transcript was subsequently cloned by a unique adaptor and loaded to a microbead. This MPSS profiling process sampled 1-2 million sequenced transcripts per library on microbeads. Sequenced tags were generated by serial cutting and ligation of decoding adaptors. Sequencing runs were done by using two different cleavage steps, which are two different four-nucleotide sequencing frames offset by two bases (two-step) or three bases (three-step) [29]. The abundance of each signature was normalized to one million (transcripts per million, TPM) for the purpose of making comparisons between samples.

### MPSS data analyses

To remove reads that might have arisen from errors in the MPSS procedure, two filters were applied to the derived reads [30]. The first, the "reliability filter," removed low quality reads containing ambiguous nucleotides or adaptor sequences. The second, the "significance filter," was intended to remove reads that were consistently present at background levels, excluding signatures lower than 3 TPM in both libraries according to the criteria described by Meyers et al. [30].

To link the expressed signatures to known genes from citrus, the unigene dataset from TIGR gene index database and Harvest database were combined to create a reference gene dataset. The signatures produced *in silico *were stored in the reference read database, through which the expressed MPSS signatures could be mapped on the corresponding EST contigs and singletons based on matches between MPSS signatures and *in silico *reads, as described previously [31]. The significance level of the differences of signature frequency and transcript abundance between the two libraries was analyzed using the Z-score method according to Kal et al. [34].

### Functional assignments of differentially expressed genes

To assign putative functions to differentially expressed genes between MT and WT, annot8r program was run locally to BLAST against a reference database that stores UniProt entries, their associated Gene Ontology (GO), Enzyme Commission (EC), and Kyoto Encyclopaedia of Genes and Genomes (KEGG) annotation [56]. The GO categorization results were expressed as three independent hierarchies for biological process, cellular component, and molecular function [57]. The biological interpretation of the differentially expressed genes was further completed by assigning them to metabolic pathways using KEGG [58]. For the identification of pathways significantly affected by the mutation, we focused on the metabolite pathways with at least three affiliated genes.

### Real-time quantitative RT-PCR verification

Thirty genes were chosen for confirmation by real-time quantitative RT-PCR with SYBR green I chemistry (QIAGEN, Germany). Primers for these genes were designed with the Primer Express software (PE Applied Biosystems, USA) and tested to ensure amplification of single discrete bands with no primer-dimers. Product size was about 180 bp. Total RNA (3 mg) was treated with 3 U of DNase (Promega, USA) and then used in first-strand synthesis with an oligo (dT) primer (20-mer) and reverse transcriptase according to the manufacturer's instructions. For real-time PCR, an amount of cDNA corresponding to 25 ng of input RNA was used in each reaction. Reactions were performed with the SYBR Green PCR Master Mix and analyzed in the ABI 7500 Real-Time System. Real-time PCR products were amplified with 1 μl of template of the RT reaction mixture, 10 μl of 2 × SYBR Green Master Mix, and 0.5 μl of forward and reverse primer (10 μmol/μl), with water to a final volume of 20 μl. The levels of gene expression were analyzed with ABI 7500 Sequence Detection System Software (PE Applied Biosystems) and normalized with the results of *β-actin*. Real-time quantitative PCR was performed in four replicates for each sample, and data were indicated as means ± SD (n = 3).

## Authors' contributions

ZJZ, AXY, HCG, and SLM are responsible for generating the MPSS data and for interpretation of the results. ZJZ and ZDL carried out the RT-PCR experiments. ZJZ drafted the manuscript. HCG and ZJZ participated in research design and statistical analyses. GWW, DXX, and HCG proposed and supervised the research. All authors read and approved the final manuscript.

## Supplementary Material

Additional file 1 **Distribution of characteristic lengths of unigenes from the MT and the WT**. Table 1: Length distribution of unigenes from the MT and the WT; Table 2: ORF length distribution of unigenes from the MT and the WT. Click here for file

Additional file 2 **Expression of unigenes in the WT**. The table lists sense and antisense expression genes of the WT. Click here for file

Additional file 3 **Expression of unigenes in the MT**. The table lists sense and antisense expression genes of MT. Click here for file

Additional file 4 **Transcription factors found in the WT and the MT**. Putative TF genes were identified by a BLAST search against rice, Arabidopsis, and Citrus sinensis TF genes downloaded from PlantTFDB (http://planttfdb.cbi.pku.edu.cn:9010/index.php). Click here for file

Additional file 5 **The biological interpretation of the differential expression genes was further completed using KEGG pathway analysis**. A total of 200 different metabolic pathways were found in this study, with some being consistent with biological processes already revealed by GO analyses. Click here for file

Additional file 6 **Primers used for real-time quantitative RT-PCR for the verification of MPSS data**. Optimal oligonucleotide sequences for real-time RT-PCR were predicted by primer express program to prevent faint PCR products as primer dimmer and false amplicon Click here for file

Additional file 7 **The sequence information of citrus genes related to flowering development**. Genes belonging to different flowering pathways are listed. Click here for file
