# Supplementary material for: Transcriptome profile analysis of flowering molecular processes of early flowering trifoliate orange mutant and the wild-type [Poncirus trifoliata (L.) Raf.] by massively parallel signature sequencing
Source: BMC Genomics. 2011 Jan 26;12:63. doi: 10.1186/1471-2164-12-63 (PMC3039610; doi:10.1186/1471-2164-12-63)
Supplement: Additional file 6 — Primers used for real-time quantitative RT-PCR for the verification of MPSS data. Optimal oligonucleotide sequences for real-time RT-PCR were predicted by primer express program to prevent faint PCR products as primer dimmer and false amplicon [file 1471-2164-12-63-S6.DOC]

**Additional data file 6**

The primer sequence information, this file lists the primers used for genes expression detection by RT-PCR.

U27895

SENSE 5-GGTCCTCCTCAATTATAGCC-3; ANTI 5-AGCATGAACAGGTGGAACAC-3

U27809

SENSE 5-GTTCCGACAACTGAGTGATA-3; ANTI 5-AGAGCCGAAACTCCTACAAC-3

U24636

SENSE 5-CCATCAATGCTTCGCCAGTT-3; ANTI 5-TCTTTGCCGTCACTCTTTCC-3

U24601

SENSE 5-TTCTTGACCCTTTCGCTGAG-3; ANTI 5-ATTGCTGTTAGGACTGTGGC-3

U1478

SENSE 5-CAACTCCTCAGCCCTTACAC-3; ANTI 5-GCCATTATTCCATGCCTTGC-3

U22804

SENSE 5-CCAAGACGGCTTTACCAGAA-3; ANTI 5-CTCAAGTCCATCCGCTCAAA-3

U20188

SENSE 5-TGGACCGCTGAGGAAGTTGA-3; ANTI 5-GCGATTTGGAAGCCGTAGAA-3

U5337

SENSE 5-TTTCAGCGGTAGCAGCAGTG-3; ANTI 5-AGGGTAGTCTTCTTCGGTGT-3

U22779

SENSE 5-TTTGAGTACGGACGAGTGGA-3; ANTI 5-AAGAGTAACGCCGAGATTGC-3

U14414

SENSE 5-CGTGTTCTTATCGGTGTTCG-3; ANTI 5-AAGTCCTCCCTCCTTGGTCT-3

U18140

SENSE 5-CCGCATCAGGAAAGCATCAT-3; ANTI 5-TAAACCACAGCTCGACCCAC-3

U11386

SENSE 5-CAAATGGCTGAGATTCCTTC-3; ANTI 5-GATTTGCTGCATCTGTCCCT-3

U28469

SENSE 5-CTCCTTACATTCACCCACTT-3; ANTI 5-GTCTTGCTCGGTTTCTTTGT-3

U28572

SENSE 5-TGTGGCAGAACAGCAGAAAG-3; ANTI 5-CGCCGTAAATGATGGAGAAG-3

U23419

SENSE 5-CATCGTCGGTATCGGCTTTC-3; ANTI 5-AGTCACGGCTTTGGCTCCTT-3

U12861

SENSE 5-GTCGTAGTAGCGGCAGTAAA-3; ANTI 5-TGACACCAACCCAGTTAGCC-3

U31613

SENSE 5-GTCACCAGCGAAACAAGGAT-3; ANTI 5-CGACCACATAAGCGTCACAA-3

U36420

SENSE 5-AATGGCAGGCTCCTCTAATC-3; ANTI 5-TTCCAGTGGTGGCATCTTGT-3

U12495

SENSE 5-GACAGCAAGTGGCGGAGGTAT-3; ANTI 5-GTAAGCCCAGCTTGAGGGAGG-3

U12163

SENSE 5-GTGCTATGTGACGCTGAAGT-3; ANTI 5-TCTCCGCATAACAATACCTC-3

U3347

SENSE 5-AAAATCTGTTGTCCGGGTAC-3; ANTI 5-TAGAGCCTTCCTTTCTGTGA-3

U19004

SENSE 5-GATGCTGAGGTTGCCGTTAT-3; ANTI 5-TCCGTTGGCTGTTGCTTGTT-3

U4356

SENSE 5-TGGGTAGAGGAAAGATGGAG-3; ANTI 5-TGGGTAGAGGAAAGATGGAG-3

U27120

SENSE 5-ACCGCTCTCAAACACATCAG-3; ANTI 5-GCAGCCTTCTCTCTCTCC-3

*LFY*

SENSE 5-AGGTCCAGAACATCGCCAAG-3; ANTI 5-TGAAAGCCCTCCTCAGTGC-3

*TFL1*

SENSE 5-GATTGTGACAGACATTCCAG-3; ANTI 5-ATGATCTCTTGATGAAGGTG-3

U27861

SENSE 5-TGATAGTCCAGCAACCACAG-3; ANTI 5-GGAGGTCCCAGATTGTAAAG-3

U12215

SENSE 5-AAGCGGCGTAGCGGATTGAT-3; ANTI 5-TGCCTTAGGTGCTCCTGAAG-3

*ACTIN*

SENSE 5-CCGACCGTATGAGCAAGGAAA-3; ANTI 5-TTCCTGTGGACAATGGATGGA-3
